# Ultrasonic Molding Technology: Recent Advances and Potential Applications in the Medical Industry

**DOI:** 10.3390/polym11040667

**Published:** 2019-04-11

**Authors:** Ulisses Heredia-Rivera, Inés Ferrer, Elisa Vázquez

**Affiliations:** 1 Tecnologico de Monterrey, Escuela de Ingeniería y Ciencias, Av. Eugenio Garza Sada #2501 Sur, Monterrey NL 64849, Mexico; ulisses.heredia@tec.mx; 2 Department of Mechanical Engineering and Civil Construction, Universitat de Girona, Av. Lluis Santalos/n, 17071 Girona, Spain; ines.iferrer@udg.edu

**Keywords:** ultrasonic molding, ultrasonic injection molding, bioresorbable polymers

## Abstract

Recently, ultrasonic molding (USM) has emerged as a promising replication technique for low and medium volume production of miniature and micro-scale parts. In a relatively short time cycle, ultrasonic molding can process a wide variety of polymeric materials without any noticeable thermal degradation into cost-effective molded parts. This research work reviews recent breakthroughs of the ultrasonic injection molding and ultrasonic compression molding process regarding the equipment and tooling development, materials processing and potential applications in the medical industry. The discussion is centered on the challenges of industrializing this technology, pointing out the need for improvement of the current process’s robustness and repeatability. Among the most important research areas that were identified are the processing of novel engineered and nanomaterials, the understanding and control of the ultrasonic plasticization process and the tooling and equipment development.

## 1. Introduction

The trend to miniaturize medical devices, the large number of minimally invasive surgeries carried out per year and the aging of the population are just several factors driving the BioMEMS market expected to reach US$7.6 billion in 2021 [1]. However, this increasing market demand requires the development of novel technologies for a low-cost, fast and flexible production of miniaturized medical devices with intricated geometries and tight tolerances.

Thermoplastic polymers have an extended use in the medical industry, not only because of economic reasons, but because they accomplish many functional features needed in medical devices, such as biocompatibility, biodegradability, corrosion resistance and tailored mechanical properties. Additionally, in the medical field, a small batch production at an affordable cost could increase the feasibility of mass customization of medical devices supporting more efficient, effective and safe treatments.

Nowadays, low-cost fabrication of thermoplastic micro-parts can be achieved by means of micro-molding techniques, such as hot embossing, injection molding, reaction injection molding, injection compression molding and thermoforming [2]. These techniques are characterized because of the use of molds and dies to replicate a specific shape on a workpiece through plastic or viscoelastic deformations [3]. Among these processes, micro-injection molding outstands as one of the most suitable ones for medium and large series of micro-structures [4]. However, small batch production of miniature/micro-parts at an affordable cost is still explored.

Despite the maturity of micro-injection molding, it faces two important challenges when processing materials for medical applications: Material degradation and waste [5]. Thermal degradation of polymers can occur, due to long heating periods in which the raw material inside the plasticization unit is exposed prior to being injected. This issue is originated because the volume of the melt in the extruder-based plasticization system is substantially bigger than the volume of the small part to be molded [6]. Additionally, a high percentage of the polymer injected into the mold is wasted on the feeding system (sprues, runners and gates) leading to inefficient use of raw materials with important cost factors, especially for medical degree [7] or engineering polymers required in high-performance applications [8].

Recently, ultrasonic molding (USM) has emerged as a promising replication technique for low and medium volume production of miniature and micro-scale parts. In a relatively short time cycle, ultrasonic molding is able to process a wide variety of polymeric materials without noticeable thermal degradation [9] and with significant energy savings [6] (no heating bands are required running consciously heating the barrel). During each molding cycle, just the required amount of raw material is ultrasonically plasticized and rapidly shaped in the mold avoiding long heating times and therefore thermal degradation. In addition, the feeding system has reduced its dimensions and can be simplified to waste less raw materials.

**Figure 1:**
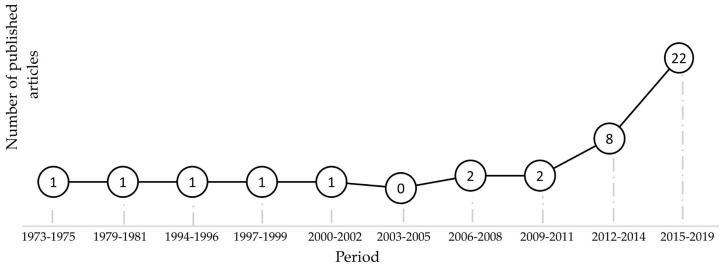
Publications in the field of ultrasonic molding technologies.

Until now, the authors have not found any dedicated article that summarizes the most important advances of this technology despite older publications in the field which are dated since 1974 [10]. Figure 1 shows that since the seventies until the turn of the century, sporadic publications were realized exhibiting a slow development of this technology. However, in the last ten years the number of publications has had a steady growth and therefore substantial research activity and interest by research groups.

A literature review could create a main framework to standardize terminology and at the same time provide an ordered and comprehensive source of information intended for new researchers in the field. Therefore, the aim of this work is to introduce the first review of this technology from the basics to the most recent research advances. Initial steps to clarify and standardize definitions related to this technology were realized. This research work also describes both, ultrasonic compression molding and ultrasonic injection molding process, including important advances in tooling, equipment development and their potential applications in the medical industry. The discussion is centered on the many challenges limiting its industrial use and research lines that could lead to significant process improvements and innovative applications in the following years.

## 2. Standardization of Terminology Used in Ultrasonic Molding

Before starting to introduce the ultrasonic molding technology, it is necessary to establish the main framework in which this review is based on. First, there are around 40 articles related to ultrasonic molding when searching through the main scientific databases considering the following keywords: Ultrasonic molding, ultrasonic micro-molding, ultrasonic plasticizing, ultrasonic micro-injection molding, and micro-ultrasonic powder molding. Next, when analyzing individual descriptions of collected articles in detail, similar and different characteristics among the processes, it was noticed that two different ultrasonic molding approaches could be classified: Ultrasonic compression molding (UCM) and ultrasonic injection molding (UIM).

The research works of Fairbanks [10], Paul and Crawford [11], Matsuoka [12], Kellomäki and Törmälä [9], Liang et al. [13] and Zeng et al. [14] were identified as ultrasonic compression molding. In all of them, the raw material is directly poured into the mold cavity, compacted by the sonotrode, and plasticized by ultrasonic energy inside the mold cavity. A combination of compacting forces and ultrasound is used to shape the powders inside the mold cavity. Thus, the material is melted and shaped in the same cavity.

On the other hand, the research works of Michaeli et al. [7,15,16], Vazquez et al. [17], Grabalosa et al. [18], Sacristan et al. [5], Diaz et al. [19], Whiteside et al. [6], Heredia et al. [20], Masato et al. [21], Sanchez-Sanchez et al. [22] and Ferrer et al. [23] were categorized as ultrasonic injection molding. In all of them, the raw material is directly poured into a plasticizing chamber prior to being injected into the mold cavity, compacted by the plunger, and plasticized by ultrasonic energy inside the plasticizing chamber (separated from the mold cavity). Next, the melt is forced to flow or injected from the plasticizing chamber into the mold cavity through a feeding system formed by runners and gates. Thus, the material is melted and shaped in different cavities. The final shape and dimensions of the molded piece are achieved by a compacting force (holding pressure) and ultrasound.

**Figure 2:**
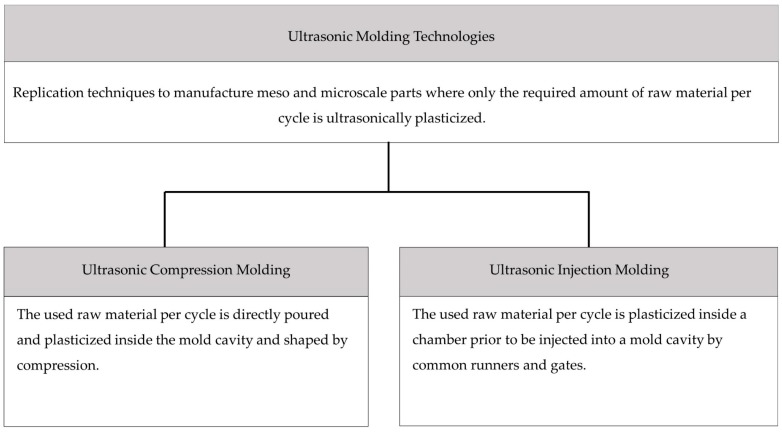
Summary ultrasonic molding technologies definitions.

However, UCM and UIM share many interesting features too. First, only the required amount of raw material per cycle is plasticized/fused; second, plasticization of the raw material is mainly produced by ultrasound energy; and finally, the dimensional range of the molded pieces and its features were around a hundred of micro-meters to a few millimeters mostly. Figure 2 summarizes the proposed definitions and classification for ultrasonic molding technologies. The shown definitions incorporate the main characteristics identified for each process, but also emphasizes their main differences. Moreover, the term ultrasonic molding is defined considering the common characteristics between UCM and UIM.

It’s worth mentioning that the ultrasonic injection molding should not be confused with ultrasonic-assisted injection molding, which uses of ultrasonic vibrations for improving replicability and filling behavior during the injection and packing phase in the injection molding process, but the raw material is conventionally melted.

## 3. Ultrasonic Compression Molding

**Figure 3:**
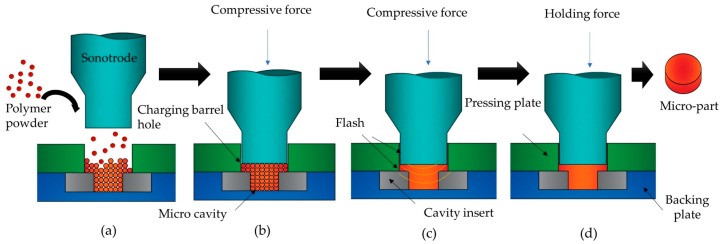
Schematic representation of the ultrasonic compression molding. (**a**) Sonotrode positioning and cavity filling; (**b**) compacting polymer powder; (**c**) ultrasonic plasticization; (**d**) cooling, release and flash peel. Adapted from Reference [25].

Ultrasonic compression molding is a manufacturing process where polymeric powders are ultrasonically plasticized and shaped by the action of compressive forces inside a mold cavity. In UCM of small parts, the polymer is rapidly plasticized and fills the mold cavity reducing problems of insufficient filling and welding marks [14]. Moreover, UCM is capable of processing polymeric and eutectic alloy powders [24] in a relatively short time cycle (usually less than 10 s). It comprises the following steps [25]. Once a mold assembly is installed and the sonotrode is well aligned with the charging barrel hole, polymeric powders are poured, filling the mold cavity and part of the charging barrel hole (Figure 3a). A precise dosage is not required because the remnant polymeric powder is pushed outwards in the form of a flash. Before applying ultrasonic vibration, polymer powders are compacted moving up and down the sonotrode until the powders inside the cavity are compacted enough (Figure 3b). Next, process parameters (sonotrode pressure, ultrasonic duration time, holding time) are set in the experimental platform and the ultrasonic vibration begins. The heat produced by the friction and deformations plasticizes the polymer powders while the micro-cavity is filled up with the molten polymer under the pressure exerted by the sonotrode. While the sonotrode moves downwards, the remaining melt is pushed upwards by a gap between the sonotrode and the charging barrel hole generating a flash (Figure 3c). Finally, the part is released, and the flash is stripped away from the part (Figure 3d).

The first UCM attempts were found in the mid-seventies when Fairbanks was able to mold at room temperature, cylindrical parts applying only ultrasound at low frequency (20 kHz) and low compacting forces. At the beginning of the eighties when Paul and Crawford [11] molded polypropylene powders in form of a tensile specimen with optimized tensile strength (20 MPa, around the 80% of values obtained with injection molding). Matsuoka [12] studied ultrasonic molding as a high-speed alternative production technique of precision products with similar mechanical strength and structure to those fabricated by traditional melting techniques. He concluded that the test specimens had a mechanical strength close to those found in conventional moldings. From the mid-seventies until the turn of the century, moldings were thick parts with overall dimensions in the mesoscale range. In terms of geometry complexity, there is evidence of simple geometric shapes, such as cylinders [10,12], circular disks [9,12], or other 2.5D geometries, such as tensile specimens. Table 1 shows examples of the dimensions and shapes of molded parts in such period. Quantitative studies about achieving tolerances and accuracy of the process were not found, Matsuoka only [12] stated that the precision of products by ultrasonic compression molding was excellent. Additional to limitations in geometrical complexity, UCM exhibited insufficient ultrasonic power to produce macroscale parts of UHMWPE with a homogenous structure [13]. This is explained because when insufficient ultrasonic power is applied to large amounts of polymeric powder (large mass) only the interface of powder particles and its surrounding is plasticized with non-recrystallized cores remaining [13].

In 2013 Liang et al. [25] introduced the use of UCM to manufacture micro-parts. They fabricated ultra-high-molecular-weight polyethylene (UHWPE) column micro-arrays, achieving good replication results reaching up to 91.9% replication rates of a micro-column of 117.3 µm of diameter. Examples of other geometric shapes recently produced are micro-dual gears of ethylene vinyl acetate with pitch diameters of 1 and 1.8 mm respectively with a 60 µm thickness [13] and polypropylene gears with pitch diameters of 4.8 mm with a 1 mm thickness [14]. Currently, this technology is focused on manufacturing micro-parts, leaving behind its application in the fabrication of macro size parts. UCM is now capable of processing miniature eutectic alloy parts [26] and composite materials (Copper-wires with a Sn-Bi matrix) [27] extending the range of its application in the near future.

### 3.1. Temperature Distribution during Ultrasonic Plasticization

**Figure 4:**
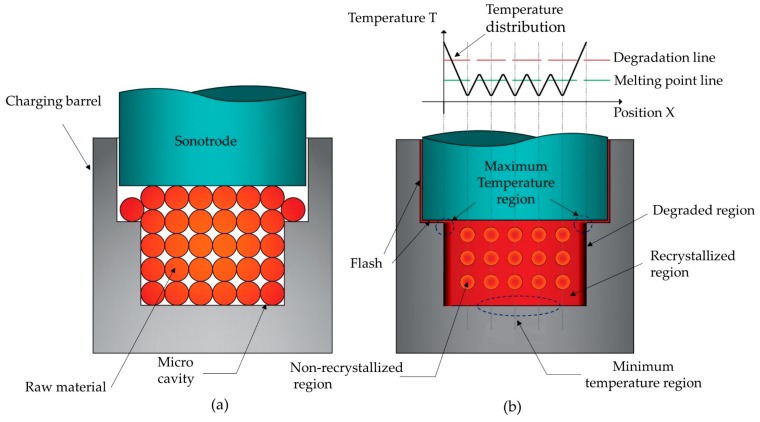
Temperature distribution in ultrasonic compression molding. (**a**) Before molding; (**b**) after molding. Adapted from Reference [13].

According to K. Zeng et al. [14] the understanding of the temperature distribution phenomenon in UCM is essential for processing homogenous and degradation-free polymeric parts. During the UCM, the heat generated by the compressive forces among the powder particles and the ultrasonic energy transmitted to the polymer increases the temperature that melts the powder particles. Large powder particles or insufficient ultrasound energy could lead to incomplete plasticization where only the surface among particles is melted and their cores remain in a solid state [26]. On the contrary, heating excess degrades the molded part. Figure 4 shows the phenomenon of distribution temperatures that occur in a UCM with incomplete plasticization. Before applying ultrasound, the polymeric powders are close to each other, but not joined or fused. During the application of ultrasound, the polymeric powders surfaces are melted but, the powder cores remain in a solid state. While the temperature is intense close to edges of the mold cavity and at the side in contact with the sonotrode, the bottom-center presented the lowest temperatures in the mold cavity. Therefore, degraded zones could appear near the edges of the mold cavity and solid-phase cores in the middle of the molded parts. This difference in the distribution of temperatures is larger in thick parts and smaller in micro-parts. However, it can be minimized or eliminated with a strict temperature control around the melting point and the polymer degradation temperature [13].

### 3.2. Equipment Development

One of the advantages of processing micro-parts by ultrasonic compression molding is the cost and simplicity of the tooling and associated equipment. So far, no commercial platform has been released to the market. In most research works a commercial ultrasonic welding machine has been adapted incorporating a mold and other fixing accessories to build a functional platform ready for molding trails. Those platforms were built for research purposes therefore the degree of automation and unattended capabilities were not developed. However, those platforms were able to control several process parameters (amplitude of vibration, ultrasound time, frequency, etc.) allowing to precisely adjust suitable combinations to process a variety of materials.

**Figure 5:**
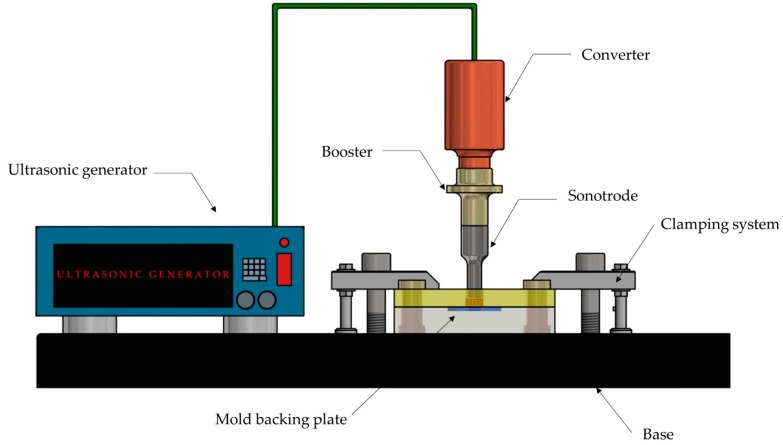
Ultrasonic compression molding platform. Adapted from [26].

An ultrasonic welding press and a mold assembly, which is shown in Figure 5, composes a basic configuration of this platform.

An ultrasonic welding machine with a 20 kHz output frequency and a 2600 W output power has demonstrated to be a suitable option to be used for UCM [24]. According to Schomburg et al. [28], ultrasonic welding machines with low operation frequencies (e.g., 20 kHz) provide more power than machines with high operation frequencies. The use of ultrasonic welding machines with more ultrasonic power would be desirable for processing big pieces and polymers with high glass transition temperatures. An ultrasonic welding machine includes an ultrasonic generator, an ultrasonic stack and an actuator. The ultrasonic generator transforms standard line power (e.g., in the US the standard line power is 120 V and 60 Hz AC) into a high frequency AC voltage, which is subsequently used as an input signal to a piezoelectric transducer or converter located within the ultrasonic stack. This high frequency signal must match the resonance frequency of the ultrasonic stack. The ultrasonic stack consists of a converter, a booster and a sonotrode or horn. All these elements are carefully designed to resonate at the specified frequency (20 kHz, 35 kHz, etc.). The converter receives the high frequency AC signal from the ultrasonic generator and converts it into mechanical vibration. This mechanical vibration is produced by the expanding and contracting piezo electric ceramics at the same frequency as the input signal. This mechanical vibration is regulated with the aid of a booster coupled with the converter and the sonotrode, either increasing or reducing the amplitude of vibrations according to the processed material. The sonotrode is used to apply the ultrasonic vibration to the raw material. Finally, a servo-driven or pneumatic-driven actuator moves linearly the ultrasonic stack toward the feedstock to be molded. It also applies the required force to mold the materials. Compared with pneumatic ultrasonic welding actuators, servo-driven actuators are more precise and repeatable. Therefore, the use of servo-driven actuators would lead to a more controlled and stable ultrasonic compression molding, improving part quality and consistency of the molded parts [29].

### 3.3. Tooling

In addition to an ultrasonic welding press, the use of molds is required to produce micro-parts by UCM. These molds carry out the encapsulation of the polymer and the expulsion of the produced micro-parts. A conventional UCM mold is composed of a mold cavity, a mold backing plate, a charging barrel hole, heating devices (optional) and a mold pressing plate. The changeable mold cavities (mold inserts) increase the flexibility of molds producing parts with similar geometries and sizes economically reusing the other components of the molds. The mold backing plate contains a pocket where the mold cavity inserts are placed and fixed. It may also have attached heating devices that are utilized for preheating the powders inside the mold cavity [24]. The mold pressing plate encloses the polymer melt inside the mold cavity. It also can hold cores or other mold cavity inserts when more complex geometries are processed. Through the charging barrel hole raw powders are pouring into the mold cavity and the sonotrode reaches the polymeric powders inside the cavity during the molding process. The mold is attached to a base with clamps or other fixing tooling avoiding collisions and misalignments. However, this mold configuration has a limited degree of automation (requiring an operator to release the moldings). Actually, UCM molds were simple in constitution fulfilling the task of encapsulating the polymers but, ejection nor any other features were incorporated; perhaps because they were exclusively used for research purposes. Additional features can be incorporated into molds to enhance their performance and process control, favoring their industrialization. For example, cooling systems enable shorter cooling times and the molded parts can be released rapidly. The use of venting or vacuum systems reduced molding problems related to trapped air and burn marks. Temperature and pressure sensors in the mold cavities provide data for optimizing the molding process and identified anomalies in quality parts, among others.

Regarding their manufacturing, it has been reported that the use of micro-staged laminated object manufacturing (DLOM), wire electrical discharged machining (WEDM) [13] and micro-drilling [25] have been used for the manufacturing of cavity inserts. However, traditional manufacturing processes, such as micro-milling, micro-EDM and micro-laser ablation could be employed for this purpose [30].

### 3.4. Materials and Processing Conditions

UCM can process a wide range of materials not limiting it only to polymer-based but also alloy-based and composite materials as well. Table 1 introduces a representative list of materials and their processing conditions reported in the literature. Red specimens represent research works where plain polymeric powders were successfully molded. Blue specimens represent research works where polymeric powders were mixed or blended with drugs (composites). Finally, gray specimens represent research works where powder alloys were fabricated by this technology.

Regarding the processing conditions in UCM, six important parameters that have effects on the final part exist. They are amplitude of vibration (A), ultrasound time (T_U_), compacting pressure (P_U_), holding time (T_H_) and mold temperature (M_T_). Moreover, the powder size and the amount of plasticized material also must be considered when processing miniature and micro-parts by UCM.

In Table 1 it is observed how short ultrasound times (T_U_) are recommended to mold small pieces and micro-features. In the polymer processing case, small amounts of feedstock are very temperature sensitive even under short periods of ultrasonic energy leading to thermal degradation if processing conditions are not set properly [5]. In the case of powder alloys, longer ultrasound times produce ultrasonic hardening reducing the formability and plasticity of the alloys. Moreover, black defects on the alloy-based part surfaces have also been reported [26]. However, not only short ultrasound times avoid thermal degradation and ultrasonic hardening but also could enhance the mechanical properties of the molded parts. Zeng et al. molded micro-parts of isotactic polypropylene with an ultrasound time of 4 s obtaining a tensile strength and elongation at break of 35.6 MPa and 193% respectively. Meanwhile, an ultrasound time of 7 s decreased both, the tensile strength and the elongation at break to around 24 MPa and 90%. Wu et al. [26] successfully manufactured small gears and mini tensile specimens demonstrating the feasibility of UCM to process Sn-Bi alloys without the need of subsequent sintering. At ultrasound time equals to 1.5 s, they obtained specimens with a tensile strength of 123.4 MPa and an elongation at break of 40.9%. Longer ultrasound times only decreased the mechanical properties of the specimens. Nevertheless, a superior hardness was achieved by increasing the ultrasonic time.

Besides the ultrasound time, the amplitude of vibration, compacting force, holding time and mold temperature can be modified to optimize the molding process. Table 1 presents a clear trend of increasing the compacting pressure as the product dimensions are reduced. Moreover, compacting pressure also influences on the flash thickness of polymeric parts scaled down when higher pressures are applied [25]. On the other hand, the effect of the amplitude of vibration in the UCM has not been fully explored. Paul and Crawford, observed that when increasing the amplitude of vibration from 84 µm to 127 µm the elongation at break and the tensile strength only were slightly improved in polyproline moldings. However, more research studied the effects of the amplitude of vibration must be conducted. About the holding time, most of the reported research works the holding time set between 3 s and 5 s (see Table 1). Holding time directly influences the cycle time therefore must be minimized to lower the production cost and at the same time, must be long enough to cool down and release the molded part without deformations or sink marks. Finally, the molding temperature has had a significant effect on the cycle time and the homogeneity part. Although the initial objective was the fabrication of moldings without additional sources of heat, it was proved that using a preheated mold, more homogenous and dense micro-parts (less and smaller micro-pores) could be achieved. Liang et al. [24] obtained single melt-recrystallized micro-columns of ultra-high molecule weight polyethylene when the mold was preheated from 25 °C to 85 °C (Table 1 reveals the processing conditions). Previous studies reported only micro-parts with a nascent and melt-recrystallized of ultra-high molecule weight polyethylene [13,25]. Considering that increasing the mold temperature could lead to prolonged cooling times, preheating must be performed at temperatures as close as possible to the environmental temperature without compromising the molding quality.

Regarding the size of powder particles, Table 1 indicates the variety of material powder size, from 50 µm to 350 µm, in average diameter. The trend seems to be that finer particle powders are preferable to manufacture low melting alloys (50 µm) and micro-features (150 µm). In fact, Liang et al. [25] employed the UCM to reproduce UHWPE column micro-arrays studying the replication rate of coarse powders (350 µm) and fine powders (80 µm). Their results demonstrated that the micro-structures of smaller dimensions obtained better replication rates with finer powders. Additionally, Paul and Crawford [11] compared the effect of powder size over mechanical properties finding that with smaller powder sizes were obtained higher tensile strength values. The finest powders reported were used to manufacture small parts of eutectic powder alloys in References [26,27].

Despite the thermal sensitivity of some bio polymers and the challenges associated to process small amounts of polymeric powders, there is evidence of successful manufacture of small parts made of an ultra-high molecule weight polyethylene, poly-l-lactides [9], polyamides [12], and polyglycolides [31] with many potential applications in the medical field which will be further discussed in Section 5.

## 4. Ultrasonic Injection Molding

**Figure 6:**
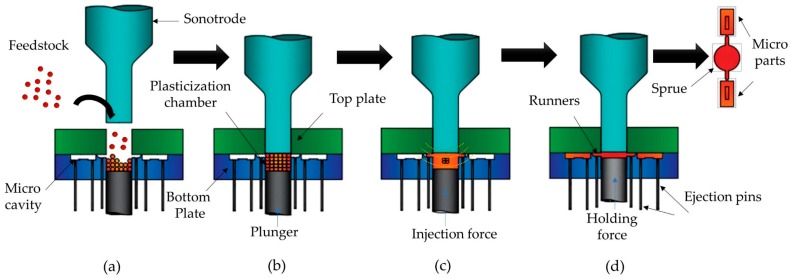
Schematic representation of the ultrasonic molding process. (**a**) Feeding; (**b**) Vibration initiation; (**c**) Plasticization and cavity filling and; (**d**) Holding stage and part release. Adapted from [18].

The ultrasonic injection molding is a manufacturing process where polymeric materials are ultrasonically plasticized and injected into a mold cavity. In terms of energy efficiency, ultrasonic injection molding is characterized by a low energy consumption for the plasticization of the polymeric materials and important reductions of material wastage [21]. Those and more characteristics made UIM a suitable process for manufacturing small and micro-parts. It comprises the following stages [18]. First, the thermoplastic material (feedstock) is introduced inside a plasticization chamber which is formed by the two halves of the mold used in the process. The feedstock geometry can be pellet-shaped, powders, thin sheets or small cylinders. This first phase is called: The feeding phase (Figure 6a). Then, the sonotrode moves down until the tip reaches the feedstock applying a pre-compression force ensuring the contact between them. Once the sonotrode is at the injection position, it vibrates with an established frequency starting the melting and plasticization of the pellets. This phase is referred to as vibration initiation (Figure 6b). While the sonotrode remains vibrating in contact with the polymer melt, the injection phase begins where the plunger moves up applying a compression force and at the same time injecting the melt through the runners until it fills the mold cavity. Therefore, the ultrasonic plasticization and cavity filling phase occur simultaneously (Figure 6c). When the mold cavity is filled up, the plunger provides a holding force to counteract polymer shrinkage and solidify the melt prior to the ejection. Finally, the sonotrode goes up to its initial position and the molded part is released by the plunger and ejector pins.

Ultrasonic injection molding has its origins with the research works of Michaeli et al. [7,15,16], they provided valuable contributions to the field. Being aware of the inefficient use of energy during melting polymer resins in screw-based plasticization units, they introduced the use of ultrasonic vibration to produce small amounts of molten plastic, which could be utilized for micro-injection molding miniature and micro-scale components [7]. Next, they adapted an ultrasonic welding press alongside a mold cavity probing the technical feasibility of manufacturing tiny parts by UIM [15]. They also conducted preliminary experiments of ultrasonically processing polyoxymethylene samples showed that less than three seconds were required to melt a mass of 500 mg. They demonstrated that contrary to UCM, feedstock geometry was not restricted to powders capable of plasticizing polymers in the form of pellets or plastic sheets. Later, Michaeli et al. [16] micro-molded different polymer resins into more complex target geometries, such as a micro-gear and a micro-tensile bar, the latter with just a 250 µm thickness.

Recently, other authors have explored the replicability and the robustness process of UIM for manufacturing challenging geometries. Heredia et al. [20] were able to fabricate thin wall plates of polylactide of 20 mm × 25 mm ×.4 mm. However, important variations in thickness were reported in parts molded with the same process parameters, these were attributed to the lack of homogeneity in size of polylactide pellets, high residual stress and premature part ejection. They proposed a stricter dosage control in the feedstock to reduce such variations. Ferrer et al. [23] studied the replicability of UIM for molding small thin wall plates of polystyrene with a micro-channel. Dimensions of the thin wall plate were 15 mm × 8 mm × 0.55 mm, while the micro-channels had 150 µm depth and 80 µm width. They found an average deviation in part thickness of less than 7% presented in the thin wall plates, with the micro-channels they had an average deviation of 4% and 11% in depth and width respectively. Nevertheless, their results on part thickness showed that 50% of the specimens were expanded, contrary to the expected shrinkage phenomenon in injection molding. The hypothesis offered was that the specimens were released too warm and relived its stored internal energy expanding their thickness. Therefore, cooling time was pointed out as a relevant parameter to increase the replication accuracy in UIM. Masato et al. [21] manufactured polypropylene micro-tensile specimens with 250 µm thickness by ultrasonic injection molding and micro-injection molding with the objective of comparing their mechanical properties and crystallization characteristics. Despite that the mechanical properties of micro-parts produced by UIM were significantly higher than those from conventionally micro-injected parts, a lower consistency in their mechanical behavior, due to a higher weight variation was identified. The high weight variation was explained by the lack of consistency and repeatability of the ultrasonic melting process. Recently, Gulcur et al. [32] molded a 5 × 5 micro-needle array, where each micro-needle had 600 µm depth and 300 µm diameter testing different feedstock geometries (pellet shaped, circular discs of 0.5 mm and 1 mm thickness) to improve the homogeneity of the ultrasonic plasticization. They found that circular discs of 1 mm thickness improved the filling behavior of the micro-needle cavities.

Besides, of the presented contributions in process robustness and replicability characteristics, in the last five years, most of the UIM contributions have been aimed to biomedical materials processing and nanocomposite materials, which are presented, in Section 4.4.

### 4.1. Ultrasonic Plasticization Mechanisms and Temperature Distribution in UIM

Most of the research in the field agree that polymer plasticization depends on two main mechanisms: (a) The interfacial friction heating and (b) the volumetric or viscoelastic heating [16,18,33,34,35,36]. The interfacial friction occurs when particles have lateral movements among them, while the damping oscillation of the polymer chains causes the viscoelastic heating, due to inner friction.

Wu et al. [33] and Jian et al. [8] studied experimentally and numerically the characteristics of the interfacial friction heating and the viscoelastic heating mechanisms in ultrasonic plasticization of polymeric granules respectively. According to Wu et al. [33] the interfacial friction phenomenon occurs only at the beginning of the ultrasonic plasticization process. It occurs in the interfaces among polymer granules ending until the interfaces disappear. Thus, it seems that the viscoelastic heating is the main heat generation mechanism in ultrasonic plasticization of polymers. Another important characteristic of interfacial friction is its relationship with processing parameters. It turns out that higher amplitudes of vibration increase significantly the interfacial friction flow rate. Additionally, the results of Jian et al. [8] research revealed that the viscoelastic heating rate is also highly influenced by the amplitude of vibration having a positive correlation. Perhaps, even more importantly is the initial temperature at which the polymer is plasticized. Plasticizing polymers with an initial temperature in the range of the glass transition temperature leads to a steep increase of the average heating rate attributed to the increased loss modulus. Therefore Jian et al. [8] recommended heating up the plasticizing chamber to the amorphous polymers glass transition temperature during ultrasonic plasticization.

Distribution of temperatures of the ultrasonic injection molding process must be evaluated during ultrasonic plasticization and during cavity filling because these two events interact and occur simultaneously. Temperature’s distribution during ultrasonic plasticization inside the plasticizing chamber is expected to be similar as in ultrasonic compression molding, previously explained in Section 3.2. On the other hand, the temperature’s distribution in the mold cavities during the cavity’s filling and the holding stage are described with the works of Whiteside et al. and Masato et al. [21]. Whiteside et al. [6] found large variations in the temperature’s distribution of consecutive filling events, suggesting that the random distribution of pellets during the ultrasonic plasticization caused this lack of repeatability during the cavity’s filling events (random hot spots generated by the interface friction heating). According to Masato et al. [21] the melt temperature flow of ultrasonic injection molding is characterized by several temperature peaks close to the gate and not on the flow front as in micro-injection molding. The continuous heat convection through the gate and the polymer is responsible for having higher temperatures at the gate and a flow front colder.

### 4.2. Equipment Development

In the beginning, all the equipment were adaptations of conventional ultrasonic welding presses built for research purposes, as described in References [15,16,37]. These systems were composed by a welding press, a mold, and an ultrasonic stack that includes three basic elements: A converter, a booster and a sonotrode (as explained in Section 3.1).

In 2013, Ultrasion S.L Company introduced to the market the first industrial ultrasonic micro-molding machine, named Sonorus 1G. It is the only ultrasonic molding machine available on the market so far. This machine operates at 30 kHz frequency with an acoustic unit which modules the amplitude of vibration delivered to the sonotrode. Additionally, it can adjust several profile injection velocities accurately. These tunable parameters enhance the flexibility and controllability of processing conditions creating more suitable combinations for the different materials to be processed.

This machine incorporates a dosing unit that delivers the equivalent mass in pellets needed to fill the sprue, runners and cavities. The dosage precision depends on the homogeneity in pellet size of feedstock because the dosage unit only feeds whole pellets. However, if more precision is required, it is also possible to weight the raw materials and feed them manually (powders or pellets). The polymer injection is realized by a plunger of 8 mm diameter which is moved by a servomotor capable of adjusting shot weights from 0.05 g to 1.5 g.

Finally, a pick and place system can take the molded pieces out the workspace demonstrating a certain degree of automation and intendant capabilities. Moreover, the small dimensions of these kinds of machines promote the development of micro-factories or in the case of medical devices, producing these components in the same hospital facilities [38].

### 4.3. Tooling

**Figure 7:**
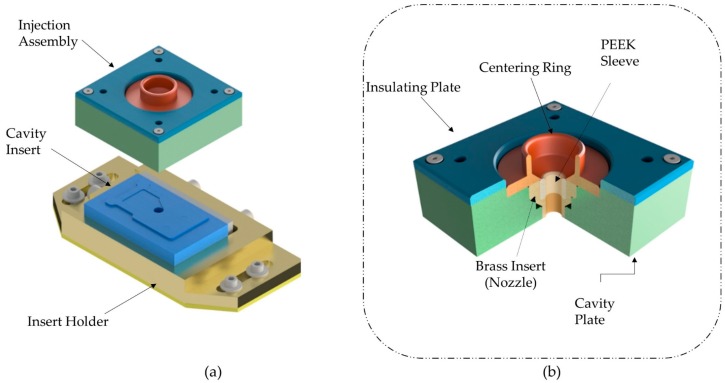
Mold assembly: (**a**) Main parts of the assembly; (**b**) an injection assembly unit. Adapted from Heredia et al. [20].

Molds or tooling for ultrasonic injection molding have been adopted from standard miniature injection molds with the only difference of the incorporation of a plasticizing chamber and a hole for pouring the raw material into the mold. Although, nowadays there is a lack of literature and investigations in tooling designs specific to this technology. Figure 7 shows a UIM mold adapted from [20]. It is mainly composed of a cavity insert, an insert holder and an injection assembly unit. The injection assembly unit is constituted of a centering ring, a PEEK sleeve and a brass insert. The centering ring has the purpose of guiding the sonotrode into the charging hole, the PEEK sleeve induces a precise alignment of the sonotrode and the plasticization chamber; the brass insert restricts the flow of the polymer melt outside of the mold. Additionally, these kinds of molds are thermally controlled to improve the process’s performance. Cooling and ejection systems are not included in this specific design for economic reasons but, their integration into the molds is desirable.

Regarding mold design, ultrasonic injection molds incorporate a feeding system, which should allow the polymer melt from the plasticization chamber to fill the mold cavities simultaneously with the same pressure and temperature. For micro-medical parts, the use of many mold cavities is not recommended because the material wastage in runners and gates could be significant and expensive. Therefore, it is desirable to consider a balance between the material wastage and the production rate.

Another important aspect of processing micro-parts is mold venting. It is well known from conventional injection molding, that air or gas presence inside the mold cavities leads to quality problems associated with short shots, burn marks and the generation of corrosive by-products that may cause serious damage in the tooling surface [39,40]. These issues become worse in micro-injection molding because inadequate air evacuation forms air pockets inside the cavity affecting part filling and replicability of the micro-structures. [41,42]. Heredia et al. [20] carried out preliminary experimental research on mold venting, where the use of venting holes improved the cavity filling of thin wall polylactide plates processed by ultrasonic injection molding. Other venting alternatives are the use of vacuum systems and the use of porous ejector pins.

Regarding mold manufacturing, Vazquez et al. [17] proposed a set of process planning considerations to improve micro-milled complex geometries in mold cavities. They successfully micro-milled a miniature acoustic guitar with tiny end mills of 100 µm diameter in a workpiece of Aluminum 7075 T6 suggesting its applicability in ultrasonic molding molds. Usually, these molds are made of aluminum and tool steels employing conventional and non-conventional machining techniques, such as micro-milling, micro-EDM and laser machining in their fabrication. Moreover, rapid tooling techniques can also be explored for the fabrication of smart and cost-effective mold inserts.

### 4.4. Materials and Processing Conditions

Recent advances in the processing of materials through ultrasonic injection molding have been focused on biomaterials and nanocomposites. Table 2 presents relevant examples of biomaterials alongside their mechanical properties and suitable process parameters. Red specimens represent research works dedicated to exploring the processability of a specific biomaterial, as well as the effect of process parameters over mechanical properties, micro-structure and other characteristics. Blue specimens are investigations where nanocomposites based on polymeric matrices were fabricated by UIM. Mechanical properties introduced in Table 2 are the maximum tensile strength (MPa) and the Elongation at break (%). Processing conditions are defined by at least five parameters which are the amplitude of vibration (A), ultrasound time (T_U_), mold temperature (M_T_), force limit (F), injection velocity (V) or as other authors call it, plunger injection profile (PVP).

Sacristan et al. [5] manufactured polylactide micro-tensile specimens made by ultrasonic injection molding and obtained that degradation depended on processing conditions, mainly ultrasonic amplitude and pressure. Low amplitudes (14.2 and 28.4 µm) resulted in more inhomogeneous polylactide specimens while more homogeneous specimens were achieved at higher amplitudes (48.1 µm). The best processing condition without degradation signals resulted using an amplitude of 48.1 µm and a molding pressure of 3 bars. Later on, Grabalosa et al. [34] studied the effect of process parameters of the ultrasonic injection molding on a part filling, mechanical properties and dimensional accuracy of polyamide parts. It was observed that complete polyamide specimens were obtained only with an amplitude of vibration over 35 µm. Ultrasonic time and pressure significantly affected on a part filling. For lower values of applied pressure, higher ultrasonic times were needed. Negre et al. [43] analyzed the processability of polypropylene specimens. As expected, drying pellets prior to molding, improved part filling, part accuracy and reduced the part porosity. Sanchez-Sanchez et al. [22] molded specimens by ultra-high molecular weight polyethylene. They found that the degree of polymer degradation was not homogenous along the specimens and increase with higher amplitude of vibration. Another important observation was that the shape of the raw material has a significant effect on the fabrication of the specimens because the formation of 80% or more complete specimens were only possible using compacted circular and compacted irregular feedstock geometries. Later, Dorf et al. [44] studied the effect of main process parameters on the mechanical properties of polyphenylsufone specimens. The highest values of tensile strength (69 MPa) were obtained with higher amplitudes of vibration (58 µm) and injection velocities (11 mm/s) combined with a short ultrasound time (1.4 s). However, all the produced samples presented polymer degradation to some degree. While some of the specimens showed visible dark marks on the surface (highly degraded parts), others had no visible defects but there was an occurrence of degradation compounds. To reduce polymer degradation, they suggested that polymers susceptible to thermal degradation must not be exposed to prolonged ultrasound times. Recently, Dorf et al. [45] were able to process polyetheretherketone specimens by ultrasonic injection molding. They used an amplitude of vibration of 58 µm and injection velocity of 6 mm/s specimens obtaining a tensile strength of 87.4–87.6 MPa, which is comparable to those from the conventional injection molding process.

UIM has not only demonstrated outstanding capacities for processing many biomaterials but as well an increasing number of articles where nanocomposites and engineered materials have been developed with UIM, expanding its potential applicability in the medical industry with the creation of smart and novel materials.

Planellas et al. [35] explored the potential of the ultrasound injection molding technology for preparing nanocomposites. They ultrasonically molded micro-tensile test specimens made of polylactide and polybutylene succinate matrices filled with four different phyllosilicates (C20A, C25A, N757 and N848) producing exfoliated clay nanocomposites. The micro-specimens revealed a relatively homogeneous dispersion of clay nanosheets in the polymer matrices oriented with the melt flow direction. According to Diaz et al. [46], high intensity ultrasonic waves are able to provide good dispersion in the preparation of polymeric matrices with nanofillers. They prepared micro-pieces composed by a biodegradable polyester matrix with functionalized silica micro and nanoparticles. Olmo et al. [47] fabricated nanocomposites, based on a biodegradable matrix of poly(ε-caprolactone) and multi-walled nanotubes (MWCNTs). Results revealed that adding MWCNTs had a significant influence on the melting and crystallization processes: Both the melting and crystallization temperatures were higher in the nanocomposite than the pure poly(ε-caprolactone) micro-parts. Recently, Sanchez-Sanchez et al. [48] demonstrated the capabilities of ultrasonic injection molding of processing ultra-high molecular weight polyethylene/graphite composites with improved mechanical properties and thermal stability.

## 5. Potential Biomedical Applications of Ultrasonic Molding Technology

As mentioned before, the main application of ultrasonic molding processes is the manufacture of small components found in many medical devices and surgical instrumentation. However, given the inherent advantages of processing a wide range of biomaterials, reduction of material wastage, low pressure molding and outstanding replication capabilities, UIM and UCM seem to be suitable processes for drug delivery, smart and tailored implants and micro-fluidic applications.

### 5.1. Drug Delivery Applications

**Figure 8:**
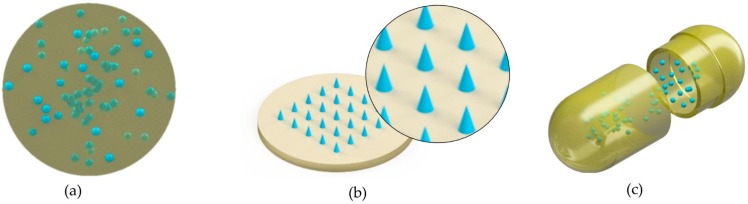
Schematic representation of different drug delivery devices. (**a**) Ultrasonically processed, solid controlled drug release devices reported in Reference [9]; (**b**) Micro-needles for transdermal drug delivery [32]; (**c**) Capsule shells for oral drug delivery [52].

An early application of ultrasonic molding technology in the medical industry was the fabrication of drug delivery devices. Kellomäki and Törmälä [9] demonstrated that through ultrasonic compression molding the decomposition of active agents and polymeric matrices was less severe compared to conventional polymer processing techniques, such as injection molding, particularly, when the polymer and the active agent (drugs) were exposed to short ultrasound times during their processing (Figure 8a). They molded miniature tablets made of different biodegradable polymers. Poly l-lactide (PLLA) tablets retained higher molecular weight compared to compression molded specimens while polylactide specimens molded by ultrasonic compression molding reached similar shear strength to those specimens molded by conventional compression molding. Moreover, poly (ortho ester) matrices that contained caffeine were successfully molded with good mechanical properties. Törmälä et al. [49] evaluated the total concentration of prepared PLLA/15 wt % levonorgestrel specimens by compression molding, injection molding and ultrasonic compression molding. While specimens processed by ultrasonic molding and compression molding exhibited levonorgestrel content close to 100% of the theoretical amount of drug, in injection molded specimens presented just 24% of the theoretical amount of levonorgestrel. Loses of drug concentration in injection molded specimens are evidence of the advantages of ultrasonically processing polymer/drug composites. Törmälä et al. [31] also processed drug/polymer matrix moldings of 66 wt % polyglycolide and 34 wt % nifedipine by UCM. Polyglycolide melting temperature (225 °C) and heat of fusion (73.6 J/g) did not change while the thermal properties of nifedipine decreased. The hydrolysis test lasted 5 weeks for the polyglycolide-nifedipine composites lowering the resorbable rate, due to the hydrophobic behavior of nifedipine within the specimens.

Another drug delivery application of ultrasonic molding would be the manufacture of micro-needles for transdermal drug delivery. In this aspect, Gulcur et al. [32] were able to mold micro-needles of polypropylene by ultrasonic injection molding (Figure 8b). Thus, a potential application of UIM would be the manufacture of dissolving micro-needles loaded with drugs, which are currently fabricated by other replication techniques, such as micro-injection molding, hot embossing and solvent casting [50]. UIM not only would lower the polymer thermal degradation but also could produce novel dissolving micro-needles for controlled biphasic drug delivery through over molding. An example of these kinds of micro-needles is given in Reference [51].

Finally, in the field of pulsatile delivery, Gazzaniga et al. [52] molded capsule shells made of Hydroxypropyl cellulose 90 wt % and polyethylene glycol 10 wt % with a nominal thickness of 300 µm, 600 µm and 900 µm by micro-injection molding (Figure 8c). However, capsule shells made from a wide variety of thermally sensitive polymers leading to the fabrication of personalized drug dosage devices by means of UIM could be a promising application. Figure 8 summarizes the three type of drug delivery devices potentially manufactured by ultrasonic molding technologies: Solid controlled drug release devices, micro-needles for transdermal drug delivery and capsule shells for oral drug delivery.

### 5.2. Smart and Tailored Implants

**Figure 9:**
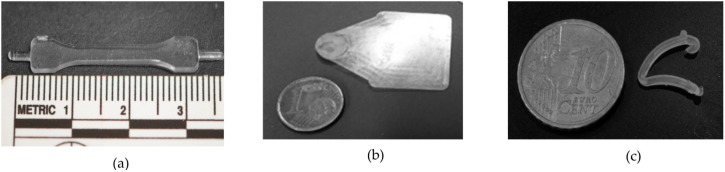
Miniature parts manufactured by ultrasonic injection molding in our facilities: (**a**) Micro-test specimen with 30 × 4 mm and 1 mm thickness; (**b**) degradable plate of 20 × 25 mm and 400 µm thickness and; (**c**) ligation system fabricated by Elias Grajeda et al. [53].

The ability of ultrasonic molding technologies to process a wide variety of resorbable and biocompatible polymers with functional improved characteristics (i.e., adding nanofillers) should be fully exploited in the manufacture of smart and tailored implants. Examples of resorbable materials shaped into challenging geometries are the research works of Heredia et al. [20] and Elias Grajeda et al. [53]. Heredia et al. [20] studied the process capabilities for fabricating miniature plates of polylactide by ultrasonic injection molding. Miniature plates have numberless applications in the medical field, such as bioresorbable plates for treating pediatric mandible fractures [54,55], craniomaxillofacial surgeries [56] or barrier devices for guided bone restoration treatments [57]. However, plates with high surface area and thin thickness require high pressures and injection velocities to avoid premature solidification of the polymer melt. Figure 9a,b show micro-test specimen and miniature polylactide plate fabricated in our facilities. In addition, Elias Grajeda et al. [53] carried out a case study where a locking ligation system made of polypropylene was used as a target geometry to demonstrate the processing capabilities of ultrasonic injection molding for manufacturing complex geometries. The miniature medical device is shown in Figure 9c. Its thickness was only 1.3 mm having a mass around 0.04 gr.

Section 4.4 introduced the research works of Planellas et al. [35], Diaz et al. [46], Olmo et al. [47] and Sanchez-Sanchez et al. [48]. All of these works are evidence of composites materials manufactured by UIM which can be employed in the fabrication of tailored implants. Moreover, it has demonstrated the overmolding capabilities of ultrasonic compression molding to create hybrid bimetallic structures [27] or a combination of metallic with polymeric materials in a single step. Although overmolding requires the use of specialized and more complex tooling, it presents interesting applications like the cost-effective assembly of polymer/metal inserts, sealing of micro-structures [58] or even the incorporation of electronic components and sensors inside the moldings.

### 5.3. Microfludic Devices and Other Medical Micro-Components

**Figure 10:**
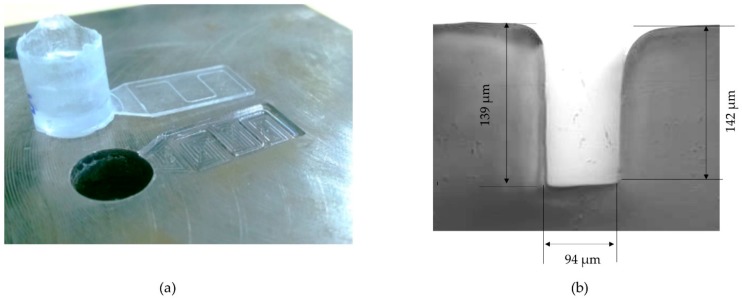
Preliminary micro-fluidic plate of polystyrene fabricated in Ferrer et al. (**a**) Micro-fluidic plate of polystyrene; (**b**) Micro-channel dimensions.

According to Sato et al. [59] the application of ultrasonic waves during the holding phase enhances the surface replication of micro-structures. Recently, Jiang et al. [60] suggested that fluidity at the micro-scale of different polymer melts generated by ultrasonic plasticization is enhanced, due to a significant reduction of shear viscosity. Despite these advantages, there are no industrial micro-fluidic devices manufactured by ultrasonic injection molding yet. Grabalosa et al. [61] and Ferrer et al. [23] demonstrated the feasibility of this technology for manufacturing micro-fluidic devices by means of a thin wall rectangular specimen of 15 × 8 × 0.55 mm which contained a micro-channel of 150 µm depth and 80 µm width. The average deviation on the part thickness was less 7% and the micro-channel accuracy was better in the depth than in width, obtaining an average deviation lower to 4% and 11% respectively. Figure 10a,b show the polystyrene specimen and the micro-channel fabricated by Ferrer et al. [23] respectively.

## 6. Discussion and Challenges

Since ultrasonic molding has been recently introduced as an alternative for the fabrication of miniature and micro-components it is evident that there are many challenges which can be raised, in addition to unifying the terminology to make easier tracking and analyzing the main contributions in the field.

Robustness and repeatability must be improved, followed by the subsequent validation of other product requirements (i.e., mechanical properties) to become an industrialized process for the manufacturing of medical devices. In fact, from the author’s perspective, the key issues rely on understanding and controlling the ultrasonic plasticization process, which is responsible to a high extent of the inherent variability in mechanical properties, replicability characteristics, dimensional accuracy and polymer degradation of molded parts.

All researches agreed that two mechanisms are present in the ultrasonic plasticization of polymers, the interfacial friction heating and the volumetric or viscoelastic heating [16]. The friction heating mechanism acts at the beginning of the ultrasonic plasticization process ending when the surface interface among powders, pellets or other feedstock shapes disappears [33]. On the other hand, the volumetric heating caused by ultrasonic energy to the polymers continues until the ultrasound source stops [8]. The most influencing factors in the ultrasonic plasticization behavior are the amplitude of vibration, ultrasound time, initial temperature of the polymer (mold temperature), shape and amount of the feedstock. Therefore, relationships among these factors and the plasticization behavior must be studied systematically for optimal control of the temperature’s homogeneity and the polymer degradation of the melt during the ultrasonic molding process.

For processing sensitive temperature bio polymers and bio materials in small amounts, the use of short ultrasound times processing is recommended to avoid polymer degradation, however, dimensional accuracy and the percentage of complete specimens could be compromised with short ultrasonic times. It is worth mentioning that the initial temperature of the polymer (mold temperature) is recommended to be above its glass transition temperature to accelerate the viscoelastic heating rate and reduce the ultrasound exposition time of polymers [8]. Interestingly, many authors use the mold temperature recommended in data sheets as the initial temperature of polymers which is not necessarily above their glass transition temperature. In injection molding for example, the plasticization unit is outside the mold and therefore the temperature in the barrel is independent of the mold temperature. Otherwise, ultrasonic compression molds and ultrasonic injection molds incorporate the plasticization chamber inside the mold causing both the mold and the initial temperature of polymers to be the same. On the other hand, the use of high mold temperatures impact on cooling time and the final quality of molded parts. Therefore, efforts should be conducted to design molds that can deal with high temperatures in the plasticization chamber and low mold temperatures for a fast part release.

Equipment development is another research line to be developed in order to industrialize this technology. This development has been characterized to be very slow with no commercial platforms for ultrasonic compression molding available in the market and only one commercial ultrasonic injection molding machine limiting their widespread and adoption by the industry and research centers. Therefore, applied research improving the performance of current and other machine configurations is required, as well as the enhancement of their current control capacities of process parameters, reduction of variability caused by environmental sources and degree of automatization. However, their simplicity in operation alongside their small dimensions are very convenient to be used in hospitals for the rapid production of customized implants, once this technology is robust and fully developed.

Tooling (mold and dies) also has shown a slow development with just a few published works about tooling design and manufacturing. In terms of mold design, most ultrasonic compression molds have functional essential elements but other features, such as cooling, vacuum and venting, variotherm even injection systems were not incorporated and studied in the performance of the process. On the other hand, the design of ultrasonic injection molds is even more challenging because the investigations of Masato et al. [21] revealed that the temperature distribution of ultrasonic molding during cavity filling is completely different from the showed in micro-injection molding. Thus, the classical design rules of the feeding system for example must be rethought to be applied in ultrasonic injection molds. Additionally, in ultrasonic injection molds several components are susceptive to wear which leads to a diminish in its replication process performance. For example, the charging hole (the inner diameter formed by the inner diameter of the PEEK sleeve and the brass insert in Figure 7) should be loose enough for a smooth movement of the sonotrode, but tight enough to avoid leaks of polymer melt causing short shots or sink marks, due to a lack of holding pressure. To avoid this kind of defects its recommended to characterize the life of this component and replace it before the wear begins to affect the part quality.

Regarding part design, the use of ultrasonic compression molding is limited to simple 2.5D geometries and typical geometries produced by conventional compression molding. Quality molded parts are also limited in thickness, because thick parts frequently suffer from partial plasticization explained by the distribution of temperatures during the process. However, ultrasonic compression molding has demonstrated outstanding replication capabilities of micro-features like the micro-column array manufactured by Liang et al. [25]. Conversely, ultrasonic injection molding can manufacture a 3D part with high complexity. The thinnest reported parts have a thickness of 250 µm [16,21] and the smallest achievable micro-features are micro-channels of 150 µm depth and 80 µm width [23]. From the author’s experience, ultrasonic injected components with a thickness of 100 µm or smaller with long lateral dimensions are highly challenging even with this technology, could require many experimental runs and high precision tooling. Nowadays there aren’t any specialized nor friendly software available in the market for ultrasonic molding simulations. Considering the complexity of the ultrasonic plasticization and ultrasonic effects on flow behavior, it is expected that simulation software for ultrasonic injection molding of micro-parts is still far away from being available for designers.

**Figure 11:**
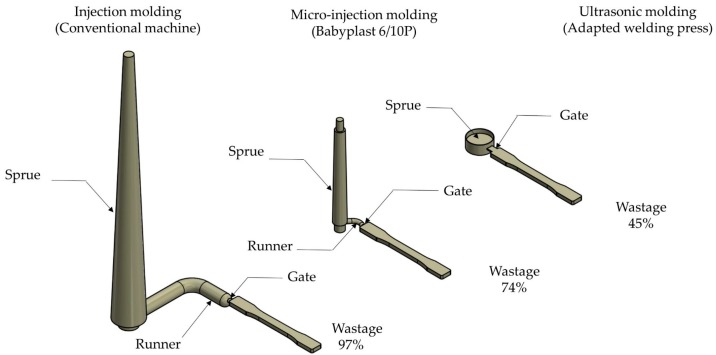
Qualitative comparison among different feeding systems (sprue + runners + gates) against ultrasonic molding sprue and runners.

Finally, one of the advantages attributed to ultrasonic molding technologies is achievable material savings. If restrictions associated to minimum shot are not considered to mold only one miniature component, a comparison of the material waste using a conventional injection molding machine, a micro-injection molding machine and an ultrasonic injection molding machine the graphical is shown in Figure 11. Dimensions of sprues and runners of a conventionally processed molding, the size of the sprue and runners provided in Reference [62] for a micro-injected component and finally for an ultrasonically processed miniature component [34] are considered to simulate the raw material waste in terms of the mass of the miniature part. Thus, in this exercise the obtained values of material wastage were 97% for the conventionally miniature molding, 74% for the micro-injected molding and 45% for the ultrasonically processed component. Avoiding raw material waste is extremely important in the production of medical devices because it directly affects the piece part price since bioabsorbable polymers can cost $5–10 USD per gram.

A summary is presented in Table 3 about the main characteristics of ultrasonic molding technologies compared to micro-injection molding, a suitable process for medical device production. This table highlights that ultrasonic technologies have a competitive advantage in terms of energy efficiency, material wastage, operability and material degradation over micro-injection molding. However, micro-injection molding is superior in categories, such as the availability of industrial equipment, process repeatability and robustness. Regarding industrial equipment the maturity and therefore the availability of many industrial machines dedicated to micro-injection molding is clear. Due to the novelty of both ultrasonic compression molding and ultrasonic injection molding, commercial equipment is still in a developing stage and is needed to double efforts from OEM manufacturers and research groups to accelerate the adoption and popularity of ultrasonic technologies by the industry. The repeatability and robustness issues of ultrasonic molding technologies are perhaps the most challenging issues to overcome and the reasons for their limited adoption by the industry. Despite expecting the reduction in the variability of ultrasonic molding technologies in the future, it is not going to substitute micro-injection molding entirely. In the mass production of medical devices micro-injection is very competitive with the largely accumulated know-how backing it up. Moreover, molded products requiring a higher percentage of elongation are more suitable for manufacture with micro-injection molding. On the other hand, ultrasonic molding technologies are more suitable for small batch production of medical devices. Among ultrasonic molding technologies, UIM could be a very promising manufacturing technology for medical device production mainly, due to its capacity of producing real 3D geometries with high precision.

## 7. Conclusions

Ultrasonic molding technologies have emerged as a promising replication technique for the low and medium volume production of miniature and micro-parts. In a relatively short cycle times, ultrasonic molding technologies can process a wide variety of polymeric materials without noticeable thermal degradation and produce cost-effective molded parts. However, there is a lack of literature about these technologies and the few articles found the use different terminology more difficult to track the latest advances in the field. Therefore, definitions of what ultrasonic molding is were proposed as a starting point to create a unified framework and standardize terminology regarding this technology. As a result, two process variants were identified: Ultrasonic compression molding and ultrasonic injection molding. Each manufacturing process was introduced, as well as the recent advances in equipment development, tooling and materials processing. Many potential medical applications were identified, such as the manufacture of drug release devices, resorbable implants and micro-fluidic devices. Despite the recent advances in the field, ultrasonic molding technologies deal with important challenges that must be surpassed to be a suitable alternative for medical device production, particularly repeatability and robustness issues of the processes. Among the most important research areas that were identified are the processing of novel engineered and nanomaterials, the understanding and control of the ultrasonic plasticization process and the tooling and equipment development research lines that could lead to significant process improvement and innovate applications in the following years.

## Figures and Tables

**Table 1:** Example of materials processed by UCM.

Product Size (mm)	Powder Size (µm)	Material	Product Weight (g)	Max. Tensile Strength (MPa)	Elongation at Break (%)	Processing Parameters	Year & Ref.
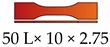	125–150	Polypropylene	1.5	20	15	P_U_ = 1.14 MPa; T_H_ = 3 s; T_U_ = 6 s; ƒ = 20 kHz; A = 127 µm	1981 [11]
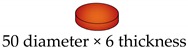	-	Polypropylene	10	28	-	P_U_ = 2 MPa; T_U_ = 5 s; ƒ = 20 kHz; A = 100 µm	1994 [12]
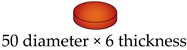	-	Polyamide	10	25	-	P_U_ = 2 MPa; T_U_ = 5 s; ƒ = 20 kHz; A = 100 µm	1994 [12]
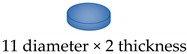	-	Polyglycolide	-	-	-	-	1996 [31]
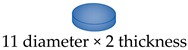	-	Poly l-lactide	-	-	-	P_U_ = 110 and 130 kPa; T_U_ = 0.17 s–0.34 s	1997 [9]
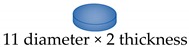	-	Poly (ortho ester)	-	-	-	P_U_ = 110 and 130 kPa; T_U_ = 0.17 s–0.34 s	1997 [9]
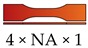	350	Isotactic Polyproline	-	35.6	193	P_U_ = 16 MPa; T_H_ = 5 s; T_U_ = 4 s; ƒ = 20 kHz	2014 [14]
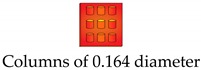	150	Ultra-high molecule weight polyethylene	-	-	-	P_U_ = 40 MPa; T_H_ = 5 s; ƒ = 20 kHz; T_U_ = 3.5 s; M_T_ = 85 °C	2015 [24]
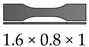	50	Sn-Bi Alloy (42% Sn–58% Bi)	-	123.4	40.9	P_U_ = 63.5 MPa; T_H_ = 3 s; T_U_ = 1.5 s; ƒ = 20 kHz	2014 [26]
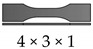	50	6 Cu-wires + Sn–Bi alloys	-	191.7	55.2	P_U_ = 63.5 MPa; T_U_ = 1.5 s; A = 34 µm; ƒ = 20 kHz	2015 [27]

**Table 2:** Examples of biomaterials processed by UIM.

Product Dimensions: Length × Width × Thickness (mm)	Feedstock Geometry	Material	Trade Name	Max. Tensile Strength (MPa)	Elongation at Break (%)	Young Modulus (MPa)	Processing Parameters	Ref.
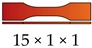	Pellets	Polylactide	Polymer 3051D	50–60	10	950 ± 40	A = 48.1 µm; P_U_ = 3 bars; T_U_ = 3 s; M_T_ = 25 °C; ƒ = 30 kHz	[5] 2013
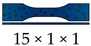	Powders	Polybutylene succinate	Bionolle 1001	75	440	320	A = 24 µm; T_U_ = 1.2s; F = 400 N; ƒ = 30 kHz	[35] 2014
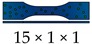	Powders	Polylactide	Ecorene NW 30	105	9.8	1660	A = 24 µm; T_U_ = 1.2s; F = 300 N; ƒ = 30 kHz	[46] 2014
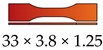	Pellets	Polyamide	Rilsamid 12G AMNO TLD	35.5–37.2	10.1–11.4	1800	A = 35 µm; P_U_ = 4 bar; T_U_ = 5 s; M_T_ = 23.5 °C; ƒ = 30 kHz	[34] 2016
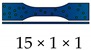	Powders	Poly(ε-caprolactone)	Solvay (128,900 g/mol Mw)	-	-	-	A = 37 µm; T_U_ = 7–8 s; F = 2500 N; ƒ = 30 kHz	[47] 2017
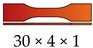	Irregular thin sheets	Ultra-high molecular weight polyethylene	Sigma-Alrich (3 × 16 g/mol Mw)	29.3	0.20	395.5	A = 56.2 µm; M_T_ = 100 °C; T_U_ = 5 s (after the first 2.5 s); V = [(2 mm/s, 5 mm), (1.5 mm/s, 5 mm), (3 mm/s, 5 mm), (6 mm/s, 2 mm)]; ƒ = 30 kHz	[22] 2017
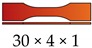	Pellets	Polyphenylsulfone	Radel R-5100 GY1137	67–70	-	-	A = 58 µm; V = 11 mm/s; T_U_ = 1.4 s; ƒ = 30 kHz	[44] 2018
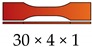	Pellets	Polyetheretherketone	KT-820 NT	87.4–87.6	-	-	A = 58 µm; T_U_ = 5 s; M_T_ = 180 °C; V = 6 mm/s constant; F = 4000 N	[45] 2018
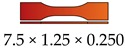	Pellets	Polypropylene	INEOS PP GA12	110–120	5–6	1000–1100	F = 700 N; T_U_ = 3 s; M_T_ = 80 °C; V = 50–200 mm/s; ƒ = 30 kHz	[21] 2018

**Table 3:** Summary of the main characteristics of ultrasonic molding technologies and its comparison with micro-injection molding.

Description	Ultrasonic Compression Molding	Ultrasonic Injection Molding	Micro Injection Molding
Industrial equipment	Not available	Sonorus 1G	Microsystem 10 & 50, Babyplast, etc.
Equipment size/cost	Small/low	Small/low	Medium/high
Production batch	Small and medium	Small and medium	Medium and high
Popularity	Very low	Low	Very High
Energy efficiency	High	High	Medium
Residence time	Short	Short	Medium to long
Repeatability and robustness	Medium	Medium	Very high
Tooling complexity	Simple	Medium	High
Part complexity	Simple (2.5D)	High (3D parts)	High (3D parts)
Flash	High	Very low	Very low
Material wastage	Low	Low	Medium
Difficulty of operation	Simple	Simple	Delicate (high-skilled operators)
Material degradation	Low	Low	Medium
Mechanical strength/ percent of elongation	High/very low	High/low	High/high
Precision of micro-features	Medium	High	High

## References

[1] Yole Developpement (2016). BioMEMS: Microsystems for Healthcare Applications.

[2] Heckele M., Schomburg W.K. (2004). Review on micro molding of thermoplastic polymers. J. Micromech. Microeng..

[3] Bissacco G., Hansen H.N., De Chiffre L. (2005). Micromilling of hardened tool steel for mould making applications. J. Mater. Process. Technol..

[4] Giboz J., Copponnex T., Mele P. (2007). Microinjection molding of thermoplastic polymers: A review. J. Micromech. Microeng..

[5] Sacristan M., Planta X., Morell M., Puiggali J. (2014). Effects of ultrasonic vibration on the micro-molding processing of polylactide. Ultrason. Sonochem..

[6] Whiteside B., Babenko M., Tuinea-Bobe C., Brown E., Coates P., Boiton P., Leach R.K., Southon N. (2016). Ultrasonic injection moulding: A study of thermal behaviour and nanofeature replication. Proceedings of the 16th International Conference of the European Society for Precision Engineering and Nanotechnology.

[7] Michaeli W., Spennemann A., Gärtner R. (2002). New plastification concepts for micro injection moulding. Microsyst. Technol..

[8] Jiang B., Peng H., Wu W., Jia Y., Zhang Y. (2016). Numerical simulation and experimental investigation of the viscoelastic heating mechanism in ultrasonic plasticizing of amorphous polymers for micro injection molding. Polymers.

[9] Kellomaki M., Tormala P. (1997). Ultrasonic moulding of bioabsorbable polymers and polymer/drug composites. J. Mater. Sci. Lett..

[10] Fairbanks H.V. (1974). Applying ultrasonics to the moulding of plastic powders. Ultrasonics.

[11] Paul D.W., Crawford R.J. (1981). Ultrasonic moulding of plastic powders. Ultrasonics.

[12] Matsuoka S. (1994). Effects of ultrasonic vibration on the compaction molding of polymeric powders. Mater. Process. Technol..

[13] Liang X., Wu X., Zeng K., Xu B., Wu S., Zhao H., Li B., Ruan S. (2014). Micro ultrasonic powder molding for semi-crystalline polymers. J. Micromech. Microeng..

[14] Zeng K., Wu X., Liang X., Xu B., Wang Y., Chen X., Cheng R., Luo F. (2014). Process and properties of micro-ultrasonic powder molding with polypropylene. Int. J. Adv. Manuf. Technol..

[15] Michaeli W., Opfermann D., Menz W., Dimov S. (2006). Ultrasonic plasticising for micro injection moulding. Proceedings of the 4 m 2006—Second International Conference on Multi-Material Micro Manufacture.

[16] Michaeli W., Kamps T., Hopmann C. (2011). Manufacturing of polymer micro parts by ultrasonic plasticization and direct injection. Microsyst. Technol..

[17] Vazquez E., Amaro A., Ciurana J., Rodriguez C.A. (2015). Process planning considerations for micromilling of mould cavities used in ultrasonic moulding technology. Precis. Eng..

[18] Grabalosa J., Ferrer I., Martinez-Romero O., Elias-Zuñiga A., Planta X., Rivillas F. (2016). Assessing a stepped sonotrode in ultrasonic molding technology. J. Mater. Process. Technol..

[19] Diaz A., Casas M., Puiggali J. (2015). Dispersion of Functionalized Silica Micro- and Nanoparticles into Poly(nonamethylene Azelate) by Ultrasonic Micro-Molding. Appl. Sci..

[20] Heredia U., Vazquez E., Ferrer I., Rodriguez C.A., Ciurana J. (2017). Feasibility of manufacturing low aspect ratio parts of PLA by ultrasonic moulding technology. Procedia Manuf..

[21] Masato D., Babenko M., Shriky B., Gough T., Lucchetta G., Whiteside B. (2018). Comparison of crystallization characteristics and mechanical properties of polypropylene processed by ultrasound and conventional micro-injection molding. Int. J. Adv. Manuf. Technol..

[22] Sanchez-Sanchez X., Hernandez-Avila M., Elizalde L.E., Martinez O., Ferrer I., Elias-Zuñiga A. (2017). Micro injection molding processing of UHMWPE using ultrasonic vibration energy. Mater. Des..

[23] Ferrer I., Vives-Mestres M., Manresa A., Garcia-Romeu M.L. (2018). Replicability of ultrasonic molding for processing thin-wall polystyrene plates with a microchannel. Materials.

[24] Liang X., Wu X., Xu B., Ma J., Liu Z., Peng T., Fu L. (2015). Phase structure development as preheating UHMWPE powder temperature changes in the micro-UPM process. J. Micromech. Microeng..

[25] Liang X., Li B., Wu X., Shi H., Zeng K., Wang Y. (2013). Micro UHMW-PE column array molded by the utilization of PCB as mold insert. Circuit World.

[26] Wu S.Y., Wu X.Y., Xu B., Cheng R., Luo F., Ruan S.C. (2014). A micro-ultrasonic powder moulding method to fabricate Sn-Bi alloy micro parts. Int. J. Adv. Manuf. Technol..

[27] Wu S., Wu X., Xu B., Chen X., Cheng R., Ruan S. (2015). Micro-structures and tensile properties of copper-wire-reinforced Sn–Bi alloy micro-composite fabricated by micro-ultrasonic powder moulding. Int. J. Adv. Manuf. Technol..

[28] Schomburg W.K., Burlage K., Gerhardy C. (2011). Ultrasonic hot embossing. Micromachines.

[29] Marcus M., Anantharaman S., Aldaz B. Advantages of a Servo-Driven Ultrasonic Welder. Proceedings of the 71st Annual Technical Conference of the Society of Plastics Engineers (ANTEC 2013).

[30] Fleischer J., Kotschenreuther J. (2007). The manufacturing of micro molds by conventional and energy-assisted processes. Int. J. Adv. Manuf. Technol..

[31] Kellomäki M., Törmälä P., Nousiainen J., Eskola H., Malmivuo J. (1996). Ultrasonic Welding-A Novel Method to Manufacture Controlled Drug Release devices. Proceedings of the 10th Nordic-Baltic Conference on Biomedical Engineering.

[32] Gulcur M., Whiteside B.R., Nair K., Babenko M., Coates P.D. (2018). Ultrasonic injection moulding of polypropylene and thermal visualisation of the process using a bespoke injection mould tool. Proceedings of the euspen’s 18th International Conference & Exhibition.

[33] Wu W., Peng H., Jia Y., Jiang B. (2017). Characteristics and mechanisms of polymer interfacial friction heating in ultrasonic plasticization for micro injection molding. Microsyst. Technol..

[34] Grabalosa J., Ferrer I., Elías-Zuñiga A., Ciurana J. (2016). Influence of processing conditions on manufacturing polyamide parts by ultrasonic molding. Mater. Des..

[35] Planellas M., Sacristan M., Rey L., Olmo C., Aymami J., Casas M.T., Del Valle L.J., Franco L., Puiggalí J. (2014). Micro-molding with ultrasonic vibration energy: New method to disperse nanoclays in polymer matrices. Ultrason. Sonochem..

[36] Jiang B.Y., Hu J.L., Li J., Liu X.C. (2012). Ultrasonic plastification speed of polymer and its influencing factors. J. Cent. South Univ..

[37] Jiang B.Y., Hu J.L., Wu W.Q., Pan S.Y. (2009). Research on the Polymer Ultrasonic Plastification. Adv. Mater. Res..

[38] Pourabdollahian G., Copani G. (2016). Toward Development of PSS-oriented Business Models for Micro-manufacturing. Procedia CIRP.

[39] Zhiltsova T.V., Oliveira M.S., Ferreira J.A. (2013). Integral approach for production of thermoplastics microparts by injection moulding. J. Mater. Sci..

[40] Griffiths C.A., Dimov S.S., Scholz S., Tosello G. (2011). Cavity Air Flow Behavior during Filling in Microinjection Molding. J. Manuf. Sci. Eng..

[41] Sha B., Dimov S., Griffiths C., Packianather M.S. (2007). Investigation of micro-injection moulding: Factors affecting the replication quality. J. Mater. Process. Technol..

[42] Sorgato M., Babenko M., Lucchetta G., Whiteside B. (2017). Investigation of the influence of vacuum venting on mould surface temperature in micro injection moulding. Int. J. Adv. Manuf. Technol..

[43] Negre P., Grabalosa J., Ferrer I., Ciurana J., Elias-Zuñiga A., Rivillas F. (2015). Study of the Ultrasonic Molding Process Parameters for Manufacturing Polypropylene Parts. Procedia Eng..

[44] Dorf T., Perkowska K., Janiszewska M., Ferrer I., Ciurana J. (2018). Effect of the main process parameters on the mechanical strength of polyphenylsulfone (PPSU) in ultrasonic micro-moulding process. Ultrason. Sonochem..

[45] Dorf T., Ferrer I., Ciurana J. (2018). Characterizing ultrasonic micro-molding process of polyetheretherketone (PEEK). Int. Polym. Proc..

[46] Diaz A., Franco I., Casas M.T., del Valle L.J., Aymami J., Olmo C., Puiggali J. (2014). Preparation of micro-molded exfoliated clay nanocomposites by means of ultrasonic technology. J. Polym. Res..

[47] Olmo C., Amestoy H., Casas M.T., Martinez J.C., Franco L., Sarasua J.R., Puiggali J. (2017). Preparation of nanocomposites of poly(Ɛ-caprolactone) and multi-walled carbon nanotubes by ultrasound micro-molding. influence of nanotubes on melting and crystallization. Polymers.

[48] Sanchez-Sanchez X., Elias-Zuñiga A., Hernandez-Avila M. (2018). Processing of ultra-high molecular weight polyethylene/graphite composites by ultrasonic injection moulding: Taguchi optimization. Ultrason. Sonochem..

[49] Törmälä P., Miettinen-Lähde S. (1997). Method for Preparing Matrix-Type Pharmaceutical Compositions through Ultrasonic Means to Accomplish Melting. U.S. Patent.

[50] Duarah S., Sharma M., Wen J. (2019). Recent advances in microneedle-based drug delivery: Special emphasis on its use in paediatric population. Eur. J. Pharm. Biopharm..

[51] Park S., Kim M., Baek S.-K., Park J.-H., Choi S.-O. (2019). Spray-Formed Layered Polymer Microneedles for Controlled Biphasic Drug Delivery. Polymers.

[52] Repka M., Reo J., Felton L., Howard S., Gazzaniga A., Cerea M., Cozzi A., Foppoli A., Maroni A., Zema L. (2011). Advanced Technologies for Oral Controlled Release A Novel Injection-Molded Capsular Device for Oral Pulsatile Delivery Based on Swellable/Erodible Polymers. AAPS PharmSciTech..

[53] Elias-Grajeda A., Garcia-Lopez E., Siller H.S., Vazquez E. (2016). Analysis and Design of a Mold to Manufacture Polymer Locking Ligation Systems by the Ultrasonic Micro-Molding Process. Master’s Thesis.

[54] An J., Jia P., Zhang Y., Gong X., Han X., He Y. (2015). Application of biodegradable plates for treating pediatric mandibular fractures. J. Craniomaxillofac. Surg..

[55] Stanton D.C., Liu F., Yu J.W., Mistretta M.C. (2014). Use of bioresorbable plating systems in paediatric mandible fractures. J. Craniomaxillofac. Surg..

[56] Schummann P., Lindhorst D., Wagner M.E., Schramm A., Gellrich N.C., Rücker M. (2013). Perspectives on resorbable osteosynthesis materials in craniomaxillofacial surgery. Pathobiology.

[57] Dimitriou R., Mataliotakis G.I., Calori G.M., Giannoudis P.V. (2012). The role of barrier membranes for guided bone regeneration and restoration of large bone defects: Current experimental and clinical evidence. BMC Med..

[58] Vingaard M., deClaville C. (2012). Sealing of polymer micro-structures by over-moulding. Int. J. Adv. Manuf. Technol..

[59] Sato A., Sakaguchi H., Ito H., Koyama K. (2010). Evaluation of replication properties on moulded surface by ultrasonic injection moulding system. Plast. Rubber Compos..

[60] Jiang B., Zou Y., Liu Y., Wu W. (2019). Characterization of the Fluidity of the Ultrasonic Plasticized Polymer Melt by Spiral Flow Testing under Micro-Scale. Polymers.

[61] Grabalosa J., Ferrer I., Negre P., Fernandez L.J., Ochoa I., Elias-Zuñiga A., Campos M., Chasoglou D. (2017). Evaluation of process parameters effects for manufacturing microfluidic devices by Ultrasonic Molding. Proceedings of the Advances in Materials and Processing Technologies (AMPT) 2015.

[62] Estrada P., Siller H.R., Vazquez E., Rodriguez C.A., Martinez-Romero O., Corona R. (2016). Micro-injection Moulding of Polymer Locking Ligation Systems. Procedia CIRP.

